# Validation of disease states in schizophrenia: comparison of cluster analysis between US and European populations

**DOI:** 10.3402/jmahp.v4.30725

**Published:** 2016-06-20

**Authors:** Katia Thokagevistk, Aurélie Millier, Leslie Lenert, Shamil Sadikhov, Santiago Moreno, Mondher Toumi

**Affiliations:** 1 Creativ-Ceutical, Paris, France; 2 Biomedical Informatics Center, Medical University of South Carolina, Charleston, SC, USA; 3 F. Hoffmann-La Roche Ltd, Basel, Switzerland; 4 Faculté de Médecine, Laboratoire de Santé Publique, Aix-Marseille University, Marseille, France

**Keywords:** schizophrenia, outcome assessment, disease states, validation, Europe, USA

## Abstract

**Background:**

There is controversy as to whether use of statistical clustering methods to identify common disease patterns in schizophrenia identifies patterns generalizable across countries.

**Objective:**

The goal of this study was to compare disease states identified in a published study (Mohr/Lenert, 2004) considering US patients to disease states in a European cohort (EuroSC) considering English, French, and German patients.

**Methods:**

Using methods paralleling those in Mohr/Lenert, we conducted a principal component analysis (PCA) on Positive and Negative Syndrome Scale items in the EuroSC data set (*n*=1,208), followed by *k*-means cluster analyses and a search for an optimal *k*. The optimal model structure was compared to Mohr/Lenert by assigning discrete severity levels to each cluster in each factor based on the cluster center. A harmonized model was created and patients were assigned to health states using both approaches; agreement rates in state assignment were then calculated.

**Results:**

Five factors accounting for 56% of total variance were obtained from PCA. These factors corresponded to positive symptoms (Factor 1), negative symptoms (Factor 2), cognitive impairment (Factor 3), hostility/aggression (Factor 4), and mood disorder (Factor 5) (as in Mohr/Lenert). The optimal number of cluster states was six. The kappa statistic (95% confidence interval) for agreement in state assignment was 0.686 (0.670–0.703).

**Conclusion:**

The patterns of schizophrenia effects identified using clustering in two different data sets were reasonably similar. Results suggest the Mohr/Lenert health state model is potentially generalizable to other populations.

Schizophrenia is a complex and multidimensional disorder that affects approximately 1% of the world's population (1) and is a leading cause of disability (2). Schizophrenia is broadly characterized by three domains of psychopathology, including negative symptoms (social withdrawal, lack of motivation, and emotional reactivity), positive symptoms (hallucinations, delusions), and cognitive deficits (working memory, attention executive function) (3).

One of the most widely used instruments to measure the severity of schizophrenia is the Positive and Negative Syndrome Scale (PANSS) (4). The scale was developed using the Brief Psychiatric Rating Scale (5) and the Psychopathology Rating Schedule (6), to provide an extensive assessment of schizophrenia symptoms. Originally, the PANSS consisted of three subscales: positive, negative, and general psychopathology (4). However, factor analyses have shown the existence of other components in the structure of the PANSS items. Despite the variety of factor models described, the five-factor model of PANSS (including negative, positive, excitement, depression, and cognitive impairment dimensions) is the most commonly reported and adopted model in the literature (7–13). Although some studies have found more factors (Van den Oord et al. (14) and Emsley et al. (15) found a six-factor and seven-factor model, respectively), there is now widespread agreement on the five-domain structure.

Factor analyses separate disease symptoms into different domains, but there are questions regarding correlations across these domains, the relative degrees of symptomatology, and whether these patterns and correlations are stable across cultures. In addition, a major concern for health economics evaluation in schizophrenia is the lack of consensus around the definition of disease states that captures the heterogeneity of symptoms.

Few studies have addressed this question of heterogeneity using cluster analytic techniques and none, to our knowledge, has compared the resulting patterns across studies and cultures. Chouinard and Albright (16) conducted a cluster analysis of end-point PANSS scores on 135 patients with chronic schizophrenia. The authors identified five clusters, but only three clusters (mild, moderate, and severe symptoms) could be evaluated due to the small sample size. Dollfus et al. (17) used Ward's method of cluster analysis on PANSS scores of 138 patients. They evaluated five subtypes of schizophrenia: positive, negative, mixed, disorganized, and schizophrenia with few symptoms. Lykouras et al. (18) also used Ward's method on PANSS scores in 255 psychiatric inpatients with schizophrenic disorder. They identified five groups of patients: the first group comprised patients with overall psychopathology of minimal severity; the second group patients with severe positive symptoms along with symptoms of psychomotor excitement; the third group patients with severe positive psychopathology only; the fourth group patients with severe positive negative depressive and cognitive symptoms; and the fifth group patients with severe negative symptoms only. These studies have limitations due to their relatively small sample sizes for clustering methods, making analyses less stable and less generalizable to other populations.

In 2004, Mohr/Lenert (19) used data from a 1-year clinical trial that collected PANSS scores and costs on 663 US patients with schizophrenia and conducted a *k*-means cluster analyses on PANSS scores for items in five-factor domains. Statistical analyses first led to a six-state framework, updated to an eight-state framework after expert review, with varying levels of positive symptoms, negative symptoms, and cognitive impairment. Lenert et al. (20, 21) published additional results, such as utility values for each of the eight disease states estimated by 620 members of the general public using the standard gamble method. Utility weights for the eight schizophrenia health states ranged from 0.44 to 0.88 and were used in several cost-effectiveness analyses evaluating treatments for schizophrenia (22, 23). While factor analyses of the PANSS showed remarkable stability of the structure across international populations (the five dimensions being positive symptoms, negative symptoms, cognitive impairment, hostility/aggression, and mood disorder), it has not been shown whether multidimensional disease states similar to those found in Mohr/Lenert would be obtained in a European population. The validation of such classification in Europe would benefit both clinicians and health economics actors, such as modelers or decision makers. Clinicians may consider these health states to classify patients based on their clinical profile, to understand disease progression, to better define the severity of patients’ symptoms, and to prescribe appropriate treatments. Health economic modelers may require a validated classification for elaborating a relevant model structure, in view of future treatment economic evaluations that will be reviewed by decision makers.

The objective of this analysis was to reassess the factor and cluster analyses performed by Mohr/Lenert (2004) and the state assignment rules developed in this cohort, using data from the European Schizophrenia Cohort (EuroSC) (24). This may be useful for defining health states based on severity of symptoms in the context of pharmacoeconomic model development.

The EuroSC participants were selected to provide a representative sample of the European population with schizophrenia. As such, the data provided a good opportunity to verify the stability of the composition of patient subgroups across two different cultures in a second large data set and to validate the use of the Mohr/Lenert classification (19) in Europe for economic analyses.

## Methods

### Data source

Data from the EuroSC (24) were used for this study (*N*=1,208). EuroSC was a naturalistic follow-up of a cohort of people aged 18–64 years, suffering from schizophrenia and in contact with secondary psychiatric services. The principle objective of the EuroSC was to identify and describe the types of treatment and methods of care for people with schizophrenia and to correlate these with clinical outcomes, states of health, and quality of life (24–31). Participants were interviewed at 6-monthly intervals for a total of 2 years, until 2002.

The study was carried out from 1998 to 2000 in nine European centers that covered France (*N*=288), Germany (*N*=618), and Britain (*N*=302). Each of these areas covered an urban center of approximately 1 million inhabitants living in a city or in medium-size towns. In each area, patients treated in the ‘psychiatric sector’ (32) were identified according to the following criteria: diagnosis of schizophrenia according to the *DSM-IV* criteria (33) and aged 18–64 years. Random sampling from these patients was used to generate a representative sample; only minor clinical and sociodemographic differences were observed between patients from the different countries (33).

This cohort was conducted in accordance with the Declaration of Helsinki and French Good Clinical Practice (34, 35). The protocol of this study was approved by the institution review board or the ethics committee responsible for the participating hospital or institution. Written informed consent was obtained from each participant after the study details had been fully explained.

### Statistical analyses

The presented statistical analyses were performed on the EuroSC data only. The results were then compared with the Mohr/Lenert (2004) data findings.

### Factor analysis

The first step was to determine the key PANSS elements that describe a domain and to verify whether the EuroSC data led to similar results as those defined in Mohr/Lenert study (2004). A principal components analysis (PCA) with Varimax rotation on standardized PANSS scores was conducted using combined visits data. Factor loadings and eigenvalues for each of these domains were provided.

### Cluster analysis

As previously performed by Mohr/Lenert (2004), the second step was to conduct a *k*-means cluster analysis on the sum of standardized PANSS scores within each of the five domains derived from the PCA. The aim of the cluster analysis was to group subjects into similar categories of disease symptoms.

The cluster analysis was performed with SAS version 9.3 proc fastclus, following MacQueen's (1967) *k*-means methodology, using an algorithm in which each item is assigned to the cluster having the nearest centroid (mean). An optimal cluster center minimizes the sum of squared distances. As the number of clusters increases, the root-mean-squared distance to the cluster center (the root-mean-squared error [RMSE]) declines.

The root-mean-squared distance was described by the number of clusters, to examine the rate of change of RMSE terms by the number of clusters.

### Description of clusters using severity of symptoms

The next step was to describe the clusters as disease states and check whether they corresponded to those derived by Mohr/Lenert (2004). Each cluster was described by three levels of severity – low, moderate or high – in terms of the first three factors accounting for positive symptoms, negative symptoms, and cognitive impairment. Levels were assigned according to cluster center values and confirmed by average domain scores within clusters.

### Association between EuroSC clusters and Mohr/Lenert disease states

The association between EuroSC and Mohr/Lenert clusters was assessed using severity levels of symptoms in the first three domains. These are presented in a contingency table (Table 4). Further description of this step is provided in the Results section.

### Agreement among the cluster assignment rules

A combined model was created that represented the synthesis of both the EuroSC clusters and the states in Mohr/Lenert model. Individual patients were assigned to these composite states based on the closest EuroSC cluster center and using the rules described for cluster assignment in Mohr/Lenert. These rules (Appendix 3 of Mohr/Lenert 2004 (19)) define cut-off points for low, moderate, and high symptoms for each of the five domains considered in the framework.

The level of agreement between the two models in assignment of individual patients to states was estimated using the Fleiss–Cohen weighted kappa statistic coefficient (36).

Statistical analyses were performed using SAS software, version 9.3.

## Results

### Factor analysis

As found in several previous studies (37) and in the Mohr/Lenert (2004) study, the PCA with Kaiser criterion conducted on EuroSC data retained five factors, accounting for 56% of total variance, to describe the structure of PANSS scores. These factors corresponded to positive symptoms (Factor 1 – eigenvalue: 4.646), negative symptoms (Factor 2 – eigenvalue: 3.954), cognitive impairment (Factor 3 – eigenvalue: 2.966), hostility/aggression (Factor 4 – eigenvalue: 2.705), and mood disorder (Factor 5 – eigenvalue: 2.679).

Table 1 presents the factor loadings of these five domains. The factor analysis was highly consistent with numerous other studies (7–13). PCA revealed the same five-factor structure, with the same items representing the factors as in the Mohr/Lenert (2004) study, except for item G12, *lack of judgment and insight*. This item has a loading value slightly greater on the positive factor than on the cognitive impairment factor (0.46 vs. 0.40).

**Table 1:** PANSS factor loadings from PCA using EuroSC data

PANSS item	Description	Factor 1 (negative)	Factor 2 (cognitive impairment)	Factor 3 (positive)	Factor 4 (hostility)	Factor 5 (mood)
G1	Somatic concern	0.0289	0.2446	0.2577	0.0530	**0.4364**
G2	Anxiety	0.1005	0.1440	0.0893	0.0866	**0.7698**
G3	Feeling of guilt	0.0922	−0.0165	0.1468	0.0200	**0.6538**
G4	Tension	0.1004	0.3308	0.0204	0.3614	**0.5752**
G5	Mannerisms and posturing	0.1969	**0.5181**	0.1627	0.1639	0.1465
G6	Depression	0.2825	−0.1173	0.0804	−0.0514	**0.6701**
G7	Motor retardation	**0.6261**	0.1833	0.0502	−0.0855	0.2088
G8	Uncooperativeness	0.2538	0.1061	0.1284	**0.7646**	−0.0282
G9	Unusual thought content	0.0885	0.2926	**0.7164**	0.1872	0.1303
G10	Disorientation	0.2214	**0.5509**	0.1181	0.1350	−0.0291
G11	Poor attention	0.2722	**0.6369**	0.1381	0.1317	0.0583
G12	Lack of judgment and insight	0.2196	0.4048	**0.4644**	0.2242	−0.0858
G13	Disturbance of volition	0.2983	**0.4914**	0.0227	0.1199	0.2328
G14	Poor impulse control	0.0596	0.3612	0.1072	**0.5689**	0.1571
G15	Preoccupation	0.3027	**0.4325**	0.2245	0.2251	0.2880
G16	Active social avoidance	**0.6107**	0.1002	0.2295	0.2361	0.3219
P1	Delusions	0.0545	0.2104	**0.8215**	0.1640	0.1905
P2	Conceptual disorganization	0.2055	**0.6764**	0.3265	0.1711	0.0836
P3	Hallucinatory behavior	0.0462	0.0359	**0.7144**	−0.0333	0.1873
P4	Excitement	−0.1494	0.4627	0.1465	**0.5013**	0.2179
P5	Grandiosity	−0.0646	0.2373	**0.4610**	0.3563	−0.0094
P6	Suspicious/persecuted	0.1616	0.0797	**0.5470**	0.4283	0.3200
P7	Hostility	0.1964	0.0982	0.2247	**0.7794**	0.0539
N1	Blunted affect	**0.7983**	0.2838	0.0261	0.0369	0.0991
N2	Emotional withdrawal	**0.8107**	0.2301	0.0708	0.1406	0.1473
N3	Poor rapport	**0.7580**	0.3128	0.0669	0.2534	0.0236
N4	Passive/apathic social withdrawal	**0.7390**	0.1806	0.1034	0.1344	0.2102
N5	Difficulty in abstract thinking	0.2937	**0.6539**	0.0782	−0.0405	−0.0424
N6	Lack of spontaneity and flow of conversation	**0.7417**	0.2955	−0.0465	0.0519	−0.0769
N7	Stereotyped thinking	0.3398	**0.5833**	0.1905	0.2059	0.1157

The factor loadings correspond to the correlation between the original variables and the factors and permit understanding of the underlying nature of a particular factor. Items in bold are used to define the domain. EuroSC, European Schizophrenia Cohort; PANSS, Positive and Negative Syndrome Scale; PCA, principal component analysis.

### k-Means cluster analysis

A *k*-means cluster analysis was conducted on the sum of standardized PANSS scores within each of the five domains. As an example, negative symptoms score was defined as the sum of *motor retardation*, *active social avoidance*, *blunted affect*, *emotional withdrawal*, *poor rapport*, *passive/apathic social withdrawal*, and *lack of spontaneity and flow of conversation* scores. Items used to define other symptom domains are presented in bold in Table 1.

**Fig. 1:**
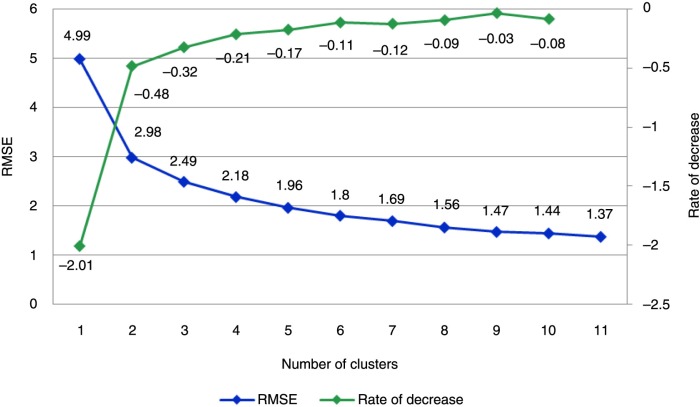
Root-mean-squared errors (RMSE) and rate of decrease by number of clusters.

RMSE and rate of decrease by number of clusters are presented in Fig. 1. As in the Mohr/Lenert (2004) study, the present cluster analysis identified two distinct subgroups of patients as the best solution for the number of disease states, as the rate of decrease of RMSE is sharply reduced at two clusters. However, when examining the curve on rate of decrease of RMSE (in green in Fig. 1), the rate of decrease increases slightly after three, four, and five clusters and then decreases after six clusters. This result suggests that there is no further improvement after six clusters. In agreement with the Mohr/Lenert (2004) study, we assumed that patients with schizophrenia may be optimally described according to six subgroups.

### Description of clusters

In this section, the described clusters were derived using the same five-factor model as in the Mohr/Lenert (2004) study.

### Characterization of clusters

Cluster center locations (standardized values) according to the first three domains (negative symptoms, positive symptoms, and cognitive impairment) are reported in Table 2. A level (low, low to moderate, moderate, moderate to high, or high) on the three domains was assigned to each cluster using the corresponding cluster center values. A low symptom level was assigned when the center value was close to the minimum value of the six clusters, a moderate symptom level was assigned when close to the value midway between the minimum and the maximum, and a high symptom level was assigned when close to the maximum. Severity levels assigned to each domain are represented by colors in Table 2.

**Table 2:** Cluster center locations on the three first domains

		Cluster center locations		
		
Cluster	*n*	Negative domain	Positive domain	Cognitive impairment domain
1	659	1.3	-0.13	0.88
2	366	1.46	1.53	1.79
3	501	-0.34	1.41	0.14
4	344	0.17	0.83	0.81
5	2,349	-0.62	-0.65	-0.69
6	633	0.3	0.09	0.03

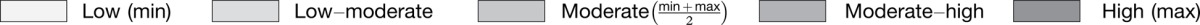

The disease severity in clusters varied from mild disease (Cluster 5), with low symptoms in all three domains, to high disease (Cluster 2), with high symptoms in all three domains. Clusters 4 and 6 were considered as moderate disease states, although both positive and cognitive symptoms were estimated as low to moderate in Cluster 6 and moderate to high in Cluster 4. The two remaining groups were both severe, with positive symptoms predominant in Cluster 3 and both negative and cognitive symptoms predominant in Cluster 1 (Table 3).

**Table 3:** PANSS five domain scores (SD) by clusters

	Mohr/Lenert classification					EuroSC finding					
		
PANSS factor	Cluster	Negative	Positive	Cognitive	Description	Cluster	Negative	Positive	Cognitive	Hostility	Mood
Contributing PANSS items	–	G7, G16, N1, N2, N3, N4, N6	G9, P1, P3, P5, P6	G5, G10, G11, G12, G13, G15, P2, N5	–	–	G7, G16, N1, N2, N3, N4, N6	G9, G12, P1, P3, P5, P6	G5, G10, G11, G13, G15, P2, N5	G8, G14, P4, P7	G1, G2, G3, G4, G6
State 1	Cluster A	<2.1 (low)	<2.7 (low)	<2.9 (low)	Mild symptoms	Cluster 5	1.42 (0.51) (low)	1.18 (0.32) (low)	1.36 (0.38) (low)	1.07 (0.18)	1.41 (0.41)
State 2	Cluster B	2.1–3.4 (moderate)	<2.7 (low)	<2.9 (low)	Moderate with negative dominance of symptoms	Cluster 6	2.36 (0.79) (moderate)	1.87 (0.72) (low–moderate)	1.95 (0.55) (low–moderate)	1.23 (0.29)	2.99 (0.57)
State 3	Part of Cluster C	<3.4 (low–moderate)	<3.9 (moderate)	<2.9 (low)	Moderate with positive and negative dominance of symptoms	Cluster 3	1.71 (0.6) (low–moderate)	3.09 (0.79) (high)	2.04 (0.56) (moderate)	1.29 (0.32)	1.86 (0.59)
State 4	Cluster D	>3.4 (high)	<3.9 (low–moderate)	<2.9 (low)	Severe with negative dominance of symptoms	Cluster 1	3.37 (0.8) (high)	1.67 (0.63) (low–moderate)	2.65 (0.66) (moderate–high)	1.31 (0.33)	1.66 (0.52)
State 5	Part of Cluster E	>3.4 (low–moderate)	<3.9 (low–moderate)	>2.9 (high)	Severe with cognitive dominance of symptoms	Cluster 4	2.23 (0.75) (moderate)	2.57 (0.86) (moderate–high)	2.58 (0.61) (moderate–high)	2.49 (0.52)	2 (0.55)
State 6	Part of Cluster C	>3.4 (high)	<3.9 (low–moderate)	>2.9 (high)	Severe with negative and cognitive dominance of symptoms	Cluster 3	1.71 (0.6) (low–moderate)	3.09 (0.79) (high)	2.04 (0.56) (moderate)	1.29 (0.32)	1.86 (0.59)
State 7	Part of Cluster E	<3.4 (low–moderate)	>3.9 (high)	–	Severe with positive dominance of symptoms	Cluster 4	2.23 (0.75) (moderate)	2.57 (0.86) (moderate–high)	2.58 (0.61) (moderate–high)	2.49 (0.52)	2 (0.55)
State 8	Cluster F	>3.4 (high)	>3.9 (high)	–	Very severe symptoms	Cluster 2	3.53 (0.81) (high)	3.23 (0.86) (high)	3.42 (0.65) (high)	2.49 (0.76)	3.04 (0.74)

Similar levels could be assigned using average domain scores (negative symptoms, positive symptoms, and cognitive impairment) on each cluster (Table 3).

### Association and agreement between EuroSC clusters and Mohr/Lenert disease states

In the Mohr/Lenert (2004) study, States 5 and 7, as well as States 3 and 6, were initially grouped together. These clusters were distinguished at a later stage after recommendations by the clinical panel.

The cross-tabulation of the Mohr/Lenert six-cluster model by the EuroSC clusters model is presented in Table 4. The majority of patients with Mohr/Lenert model State 1 (mild) corresponded to Cluster 5 in the EuroSC model (83.4%). The majority of patients with Mohr/Lenert model State 8 (very severe) corresponded to Cluster 2 in the EuroSC model (93.2%). EuroSC Cluster 6 could be assigned to Mohr/Lenert State 2 (named *Cluster B* in Table 3) as moderate with negative dominance of symptoms. Similarly, EuroSC Cluster 1 could be assigned to Mohr/Lenert State 4 (Cluster D) as severe with negative dominance of symptoms.

**Table 4:** Cross-tabulation of Mohr/Lenert six-cluster model with the EuroSC cluster model

	EuroSC cluster model [Number of patients (combined visit data) and row percentages]						
	
Mohr/Lenert state model	1	2	3	4	5	6	Total
1 (Cluster A)	7 (0.3%)	0 (0%)	114 (4.85%)	51 (2.17%)	**1,962 (83.42%)**	218 (9.27%)	2,352 (100%)
2 (Cluster B)	243 (26.67%)	15 (1.65%)	33 (3.62%)	86 (9.44%)	294 (32.27%)	**240 (26.34%)**	911 (100%)
3–6 (Cluster C)	115 (18.40%)	140 (22.40%)	**217 (34.72%)**	85 (13.60%)	0 (0%)	68 (10.88%)	625 (100%)
4 (Cluster D)	**181 (60.13%)**	46 (15.28%)	2 (0.66%)	11 (3.65%)	0 (0%)	61 (20.27%)	301 (100%)
5–7 (Cluster E)	86 (20.38%)	115 (27.25%)	96 (22.75%)	**93 (22.04%)**	3 (0.71%)	29 (6.87%)	422 (100%)
8 (Cluster F)	1 (2.27%)	**41 (93.18%)**	2 (4.55%)	0 (0%)	0 (0%)	0 (0%)	44 (100%)
Total	633 (13.60%)	357 (7.67%)	464 (9.97%)	326 (7.00%)	2,259 (48.53%)	616 (13.23%)	4,655 (100%)

Bold and grey cells correspond to the assignment within both disease sets (higher row percentage).

The remaining EuroSC Clusters 3 and 4 were both severe. As levels on positive and cognitive symptoms in EuroSC Cluster 4 ranged from moderate to high, Cluster 4 was assigned to Mohr/Lenert Cluster E (combining States 5 and 7). EuroSC Cluster 3 was then assigned to Mohr/Lenert Cluster C (combining States 3 and 6) (Table 3).

We then assigned individual patients to these composite model states based on the two assignment rules. The resulting Fleiss–Cohen weighted kappa coefficient (95% confidence interval) was estimated at 0.686 (0.670–0.703).

## Discussion

Developing models that map disease-specific measures such as the PANSS to health states for the purpose of economic evaluation, for example, is a difficult task. The intent of such cluster analysis is to individualize homogeneous clusters of patients whose disease might be determined by biological process, differing from one group to another.

The approach proposed by Mohr/Lenert considered ‘big data’ statistical methods to identify disease states in schizophrenia through cluster analysis. These disease states were subsequently converted to health states (full descriptions of the quality of life of the individual) for value rating tasks. The question posed by reviewers at the time of publication of the two paired papers (19, 20), and today, is whether clustering or statistical methods that use natural covariance to define disease states produce disease states that are generalizable to other populations or merely statistical summaries of the data from one trial. That is to say, can the disease states identified in one trial be *reused* in others as part of a mapping function?

Using data from the EuroSC, a 2-year observational study of 1,208 patients with schizophrenia, the same clustering method as in the Mohr/Lenert (2004) study was used to verify the stability of the structure and the multidimensional disease states across international populations. Although the EuroSC cohort was conducted about 15 years ago, the evaluation of the severity of symptoms, as assessed by the PANSS, has not changed. Patients still have negative symptoms, positive symptoms, or cognitive symptoms and therefore such old data may not be considered as a limitation.

As previously revealed (37) with various populations and cultures, the present factor analysis suggests that dimensions of schizophrenia as measured by the PANSS are well represented by a five-factor structure, corresponding to positive symptoms, negative symptoms, cognitive impairment, mood disorder, and hostility/aggression. The resulting EuroSC five-factor model is similar to the Mohr/Lenert (2004) findings, with the same items representing the factors, except for item number G12 (*lack of judgment and insight*), which presented a loading value slightly greater on the positive factor than on the cognitive impairment factor (0.46 vs. 0.40).

The cluster analysis is a way of looking at how covariance of symptoms among dimensions in the PANSS is manifest. As in the Mohr/Lenert (2004) study, the present *k*-means analysis identified six distinct disease state clusters among patients suffering from schizophrenia and in contact with secondary psychiatric services in Europe.

One limitation of the Mohr/Lenert disease states is that they were defined only according to the first three principal component domains identified and did not account for variability in the mood disorders and hostility/aggression seen in schizophrenia. This is a limitation as the presence of mood disorders or hostility/aggression are considered as major treatment outcomes by some clinicians and should be used for the definition of disease states. However, Mohr/Lenert (2004) justified this decision, stating that the addition of two more domains would have tripled the number of disease states, even if only two levels were used to describe these domains, making preference rating of the states and application of the model to clinical trial data impractical. Our analysis parallels the Mohr/Lenert (2004) approach. However, understanding which states are comparable with each other in two different analyses over five separate domains might be difficult. To verify whether the cluster analysis on EuroSC resulted in disease states similar to the Mohr/Lenert (2004) study, each cluster was described by its corresponding severity level within each of the positive symptoms, negative symptoms, and cognitive impairment domains.

The description of clusters by their corresponding severity level (low, moderate, severe) of positive symptoms, negative symptoms, and cognitive impairment was assessed using the cluster center locations on the domains. This approach may be discussed, but when describing the average scores of negative, positive, and cognitive symptoms across clusters, it was verified that the scores increased with the attributed severity level.

EuroSC clusters were assigned to Mohr/Lenert states using the associated levels of positive symptoms, negative symptoms, and cognitive impairment. It should be noted that the EuroSC clusters model did not fit the Mohr/Lenert clusters model perfectly, probably due to differences in the range of symptom severity in the two populations. In particular, EuroSC Cluster 3 was assigned, with high positive symptoms, to Mohr/Lenert States 3 and 6, with low-to-moderate positive symptoms. However, when we aligned EuroSC Cluster 3 with Mohr/Lenert States 5 and 7 and aligned Cluster 4 with Mohr/Lenert States 3 and 6, substantial or good agreement in state assignment between the EuroSC model and the Mohr/Lenert assignment model was estimated (Fleiss–Cohen's kappa coefficient at 0.665 [0.648–0.682], interpreted using the commonly cited scale (38)). Some variability should be expected, given the differences in populations in the two studies, cultures, and differences in training of the raters measuring PANSS scores.

An advantage of having cluster definition rather than only dimension scores is that participants can be classified with several types of symptoms. Although a lot of studies use dimensional scores (positive and negative subscores of PANSS), clusters with a possibility of mixed profiles may provide a better understanding of the disease. Indeed, it is widely recognized that the number of factors used when considering the PANSS could influence the identification of subtypes of schizophrenia and/or the psychopathological processes underlying them, which may influence prognosis, therapeutic approaches, response to treatment, and prediction of related variables (8, 14, 39–41).

The strengths of this study include the use of a large highly generalizable cohort of patients with schizophrenia and the use of a validated statistical method, particularly appropriate to highlight the heterogeneity in schizophrenia. Indeed, *k*-means clustering approach ensures minimal variation within the clusters but a maximal variation among the clusters, creating homogeneous subgroups.

This study also has a number of limitations. Most of them are shared with the Mohr/Lenert study (2004), as the quantitative analysis used in both studies was similar. Moreover, there is some question of the validity of the PANSS rating in the real world *versus* randomized clinical trials. In addition, it is noteworthy to mention that EuroSC considered patients from the United Kingdom, France, and Germany, but none from late entrants in Europe, with less advanced healthcare. Therefore, the generalizability of such clusters should be taken with caution in such cases. In conclusion, this study compared two sets of disease states for schizophrenia using the PANSS derived in the Mohr/Lenert study (2004) and in the EuroSC study. Both the factor structure and the number of discrete clusters required to explain variation in symptom levels in empirical models were similar in US and European populations. The resulting substantial agreement in assignment suggests that disease states obtained using *k*-means clustering from the PANSS are comparable between US and European populations, as are the state assignment rules developed from using each data set. The present findings provide additional support for the validation of the Mohr/Lenert classification and confirm that symptom severity in schizophrenia is characterized by heterogeneity and that similar patterns of heterogeneity exist in two different data sets across two different cultures. Further study is needed to determine if the patterns reflect different phenotypes of the disease, and investigation into the biological basis for symptom clustering should be explored.
